# High-throughput sequencing of small RNAs and analysis of differentially expressed microRNAs associated with high-fat diet-induced hepatic insulin resistance in mice

**DOI:** 10.1186/s12263-019-0630-1

**Published:** 2019-02-19

**Authors:** Xue Zhao, Zhao Chen, Zengyuan Zhou, Yuzheng Li, Yuanyuan Wang, Zihao Zhou, Huimin Lu, Changhao Sun, Xia Chu

**Affiliations:** Department of Nutrition and Food Hygiene, Public Health College, Harbin Medical University, 157 Baojian Road, Nangang District, Harbin, 150081 Heilongjiang People’s Republic of China

**Keywords:** High-throughput sequencing, MiRNAs, Hepatic insulin resistance

## Abstract

**Background:**

Hepatic insulin resistance (IR) plays a crucial role in the development of many metabolic diseases, such as type 2 diabetes. MicroRNAs (miRNAs) are involved in the pathogenesis of IR and related diseases; however, studies of miRNAs in hepatic IR are limited.

**Method:**

In this study, we adopted a high-throughput sequencing approach to construct small RNA libraries in the livers of normal mice and high-fat diet-induced hepatic IR mice.

**Results:**

Through analysis of data, 107 known and 56 novel miRNAs were identified as differentially expressed miRNAs between the two groups. Additionally, bioinformatics methods were used to predict targets of the differentially expressed miRNAs and to explore the potential downstream Gene Ontology categories and Kyoto Encyclopedia of Genes and Genomes pathways. Meanwhile, some differentially expressed miRNAs (*miR-34a-5p*, *miR-149-5p*, *miR-335-3p*, *miR-10b-5p*, *miR-1a-3p*, *miR-411-5p*, and *miR-592-5p*) were validated by quantitative-time PCR, and their potential target genes related to IR or glycolipid metabolism were also predicted and presented in this study.

**Conclusion:**

Taken together, our results defined miRNA expression signature that may lead to hepatic IR in mice, and the findings provided a foundation for future studies to further explore the effects and underlying mechanisms of the miRNAs and their target genes in the pathogenesis of hepatic IR and related diseases.

**Electronic supplementary material:**

The online version of this article (10.1186/s12263-019-0630-1) contains supplementary material, which is available to authorized users.

## Introduction

Insulin resistance (IR) is thought to be a major etiological factor in the development of some metabolism-related diseases, such as type 2 diabetes. As is well known, the liver is not only vital tissues regulating glucose and lipid metabolism but a main target organ for insulin action in the body. Under normal conditions, insulin reduces liver glucose production through increasing glycogenesis and decreasing hepatic gluconeogenesis and glycogenolysis, thereby maintaining plasma glucose concentrations within a certain range. Hepatic IR causes the increase in hepatic glucose production, which contributes to the development of hyperglycemia of type 2 diabetes [1]. Thus, hepatic IR is a hallmark of type 2 diabetes.

The occurrence and development of hepatic IR is quite complex and influenced by various factors [2]. For instance, nutrient overload or physical inactivity may induce a network of oxidative stress, inflammasome activation, and autophagy that jointly inhibit insulin signaling in hepatocytes [3]. However, the pathogenesis of hepatic IR remains inadequately understood. MicroRNAs (miRNAs) are endogenous noncoding RNAs that are approximately 22 nucleotides in length, which can regulate target gene expression at the post-transcriptional level by specifically binding to the 3′-untranslated region (3′UTR) of the target mRNAs [4]. MiRNAs are involved in numerous biological processes, such as developmental, cell differentiation, and disease processes. In particular, miRNAs have been found to play an important role in IR and related diseases [5–8]. For instance, the overexpression of *miR-29* in type 2 diabetes influences glucose and lipid metabolism in skeletal muscle [7]. Although miRNAs have emerged as important regulators of glucose and lipid metabolism, miRNAs specifically involved in the progression of hepatic IR are poorly characterized.

At present, there is sufficient evidence that hepatic IR can be induced by long-time high-fat diet (HFD) [9]. Therefore, in this study, in order to provide basic information for definition and identification of miRNAs associated with hepatic IR, high-throughput sequencing of small RNAs was performed and analyzed in the livers of normal diet (ND) mice and HFD-induced hepatic IR mice. Differentially expressed miRNAs were obtained through analysis and comparison. Afterwards, the potential target genes of these differentially expressed miRNAs were predicted, and relevant pathways were analyzed. Meanwhile, some miRNAs were selected and validated by quantitative real-time PCR (q-PCR), and their potential target genes relative to IR or glycolipid metabolism were displayed in our study. Overall, our findings provide a global view of specific miRNA expression profiles in the liver of HFD-induced hepatic IR mice, which is expected to contribute to future studies of miRNAs and their target genes’ regulatory mechanisms in the pathogenesis of hepatic IR and related diseases.

## Material and methods

### Animals and diet

Male 8-week-old C57BL/6J mice were purchased from Vital River Laboratory Animal Technology (Beijing, China). All of the mice were housed in a pathogen-free barrier facility at a temperature of 22 ± 2 °C and maintained on a 12-h light/dark cycle with water ad libitum. After 1 week of adaptive feeding, mice were randomly assigned to either a normal diet (ND, *n* = 12) group or a high-fat diet (HFD, n = 12) group. Diet formula is showed in Additional file 1. The mice were fed for 12 weeks. Food intake and body weight were measured daily and weekly, respectively. At the end of the 12th week of the feeding experiment, five mice randomly selected from each group were sacrificed for blood and liver tissue collection.

### Intraperitoneal glucose tolerance test (IPGTT)

After a 12-week feeding trial, the remaining mice (ND, *n* = 7; HFD, *n* = 7) were fasted overnight, and they were administered glucose (2 g/kg body weight) by intraperitoneal injection. Blood samples were collected from the tail tip at 0, 30, 60, 90, and 120 min. Blood glucose was measured with an Accu-Chek Performa glucometer (Roche Diagnostics GmbH, Mannheim, Germany).

### Serum lipids, insulin, and homeostasis model assessment (HOMA)-IR

Serum triglyceride (TG), total cholesterol (TC), high-density lipoprotein cholesterol (HDL-c), and low-density lipoprotein cholesterol (LDL-c) were examined using kits purchased from Biosino Biotechnology Co. (Beijing, China). Mouse insulin was measured with a rat/mouse insulin ELISA kit (LINCO Research, St Charles, MO, USA). HOMA-IR was calculated using the following formula: fasting glucose (mmol/L) × fasting insulin (mU/L)/22.5.

### Western blotting

Western blotting was carried out as previously described [10]. The primary antibodies for the protein kinase B (Akt), phosphorylated Akt (p-Akt), glycogen synthase (GS), phosphorylated GS (p-GS), and phosphoenolpyruvate carboxykinase 1 (PCK-1) were obtained from Cell Signaling Technology (Beverly, MA, USA), and the primary antibody for glucose-6-phosphatase catalytic subunit (G6PC) was purchased from Santa Cruz Biotechnology Inc. (Santa Cruz, CA, USA). Second antibody was goat anti-rabbit IgG from Santa Cruz Biotechnology Inc. (Santa Cruz, CA, USA). A representative blot was shown in the figures, and each test was performed at least three times.

### Oil Red O staining

The excised livers were rapidly fixed in 10% neutral-buffered formalin solution for 24 h. And liver tissues were then embedded in paraffin, cut into 5-μm-thick sections, and stained with Oil Red O. The digital images of Oil Red O staining of livers were captured using a Nikon Eclipse Ti-S microscope (Nikon, Tokyo, Japan).

### Small RNA library construction and sequencing

Total RNA from the liver of ten male mice (ND, *n* = 5; HFD, *n* = 5) was extracted using TRIzol® Reagent (Invitrogen, Carlsbad, CA, USA) following the manufacturer’s instructions. And high-throughput sequencing was performed in the liver tissues of each mouse individually (Majorbio BioPham Technology Co., Shanghai, China). Briefly, two small RNA libraries specifically from five ND-fed mice and five HFD-fed mice were generated using the Truseq™ small RNA sample preparation Kit (Illumina, San Diego, CA, USA), and the small RNA sequencing was performed using HiSeq 4000 SBS Kit (Illumina, San Diego, CA, USA).

### Sequencing data analysis

The raw reads from high-throughput sequencing were performed with quality control to result in clean reads. The basal quality control was that of trimming low-quality reads (ambiguous N), adapter sequences, sequences < 18 nt, and sequences > 32 nt. And the clean reads were obtained and calculated for their length distributions using Fastx-Toolkit. And the identical reads were collapsed to obtain the unique reads for the analysis of the small RNA’s species and abundance. In addition, the clean reads were blasted against the Rfam database and GenBank noncoding RNA database to annotate the miscellaneous RNAs. After filtering out the rRNA, snoRNA, snRNA, tRNA, the remaining small RNAs’ distributions were analyzed on genome and their expressive quantity was calculated using Bowtie [11]. In addition, the known miRNAs and the novel miRNAs were identified and predicted using miRBase 21.0 and miRDeep2 [12], respectively.

### Analysis of differentially expressed miRNAs

Differentially expressed miRNAs were identified using TPM (transcripts per million clean tags) and DESeq2 software [13, 14]. TPM was applied for homogenization of miRNA expression, and the values were calculated using the following equation: TPM =$\frac{\mathrm{miR}\_\mathrm{readscounts}\times \mathrm{1,000,000}}{\mathrm{librarysize}}$. Following normalization, the value was set as 0.01 by default when the sequencing read was zero. After normalization, differentially expressed miRNAs were established at log_2_ (HFD/ND) ≥ 1 and 푃 value ≤ 0.05. And scatter plots and volcano plots were generated to visualize the differentially expressed miRNAs between ND and HFD mice.

### Prediction and functional analysis of target genes of differentially expressed miRNAs

Target genes of differentially expressed miRNAs were predicted using miRanda [15]. The functional categories and pathways of the target genes were analyzed by the Gene Ontology (GO) category [16] and Kyoto Encyclopedia of Genes and Genome (KEGG) pathway database, using Goatools and KOBAS [17], respectively. The threshold (*P* value ≤ 0.05 and false discovery rate ≤ 0.05) was calculated using Fisher’s exact test.

### Quantitative real-time PCR

The total RNA with miRNAs was isolated from the livers of ten mice (ND, *n* = 5; HFD, *n* = 5) by miRNeasy Mini Kit (Qiagen, Hilden, Germany) according to the manufacturer’s instructions. Reverse transcription of miRNAs was performed using miScript II RT Kit (Qiagen, Hilden, Germany). Real-time PCR was performed with the miScript SYBR® Green PCR Kit (Qiagen, Hilden, Germany) using a 7500 FAST real-time PCR system (Applied Biosystems, Foster City, CA, USA). The expression of U6 was used as an internal control, and three biologically independent replicates were analyzed for all reactions. Details of the primers used for q-PCR of miRNAs are showed in Additional file 2.

### Statistical analysis

All data were analyzed by SPSS 21.0 (Beijing Stats Data Mining Co. Ltd., Beijing, China). Two-tailed Student’s *t* test was performed to analyze differences between two groups, and *P* < 0.05 was considered statistically significant.

## Results

### Characterization of HFD-induced hepatic IR in mice

**Fig. 1:**
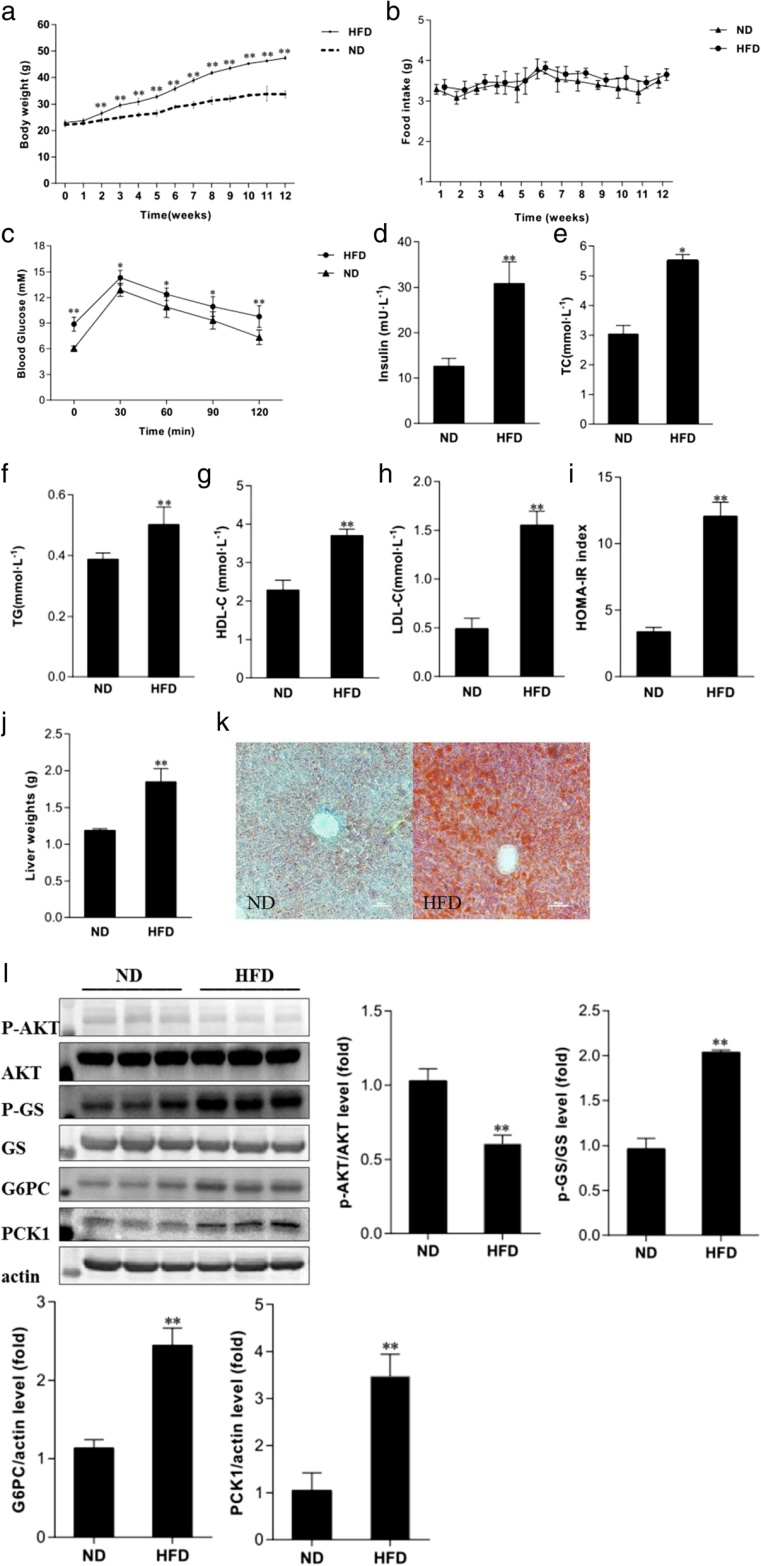
Metabolic features of mice. **a**, **b** The dynamic changes in body weights (**a**) and food intake (**b**) of mice fed ND and HFD for 12 weeks. **c** The dynamic changes in blood glucose in mice during IPGTT. **d**–**l** The changes in serum insulin (**d**), TC (**e**), TG (**f**), HDL-C (**g**), LDL-C (**h**), HOMA-IR (**i**), liver weights (**j**), liver TG content (Oil Red O staining) (**k**), and the expression of p-Akt, Akt, p-GS, GS, G6PC, PCK1, and β-actin proteins (**l**) in liver tissues in the two groups of mice. ND, normal diet mice; HFD, high-fat diet mice; *P < 0.05, **P < 0.01, compared with the value of the ND group

At the beginning of the experiment, there was no statistically significant difference in body weight of mice between the two diet groups. After HFD feeding, the body weight in the HFD group was gradually higher than that in the ND group, although there was no significantly difference in food intake between the two groups (Fig. 1a, b). At the end of the 12th week, serum glucose, insulin, TG, TC, HDL-c, and LDL-c as well as HOMA-IR were significantly higher in the HFD group, compared with those in the ND group (Fig. 1c–i). Moreover, HFD mice exhibited a hyperglycemic response to glucose loading in IPGTT, suggesting impaired glucose tolerance (Fig. 1c). Besides, liver weight and TG content in HFD mice were significantly higher than those in ND mice (Fig. 1j, k). Furthermore, compared with the ND group, the expressions of p-GS, G6PC, and PCK1 proteins were significantly increased, and the level of p-Akt protein was significantly decreased in the liver of HFD mice (Fig. 1l), indicating the presence of hepatic IR in HFD mice.

### Construction of small RNA libraries by high-throughput sequencing

**Fig. 2:**
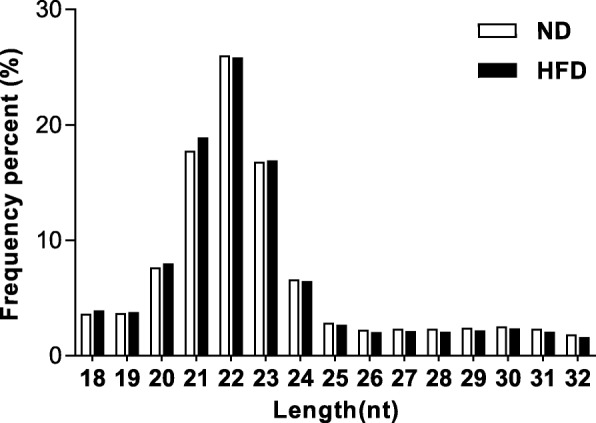
The length-frequency distribution of clean reads in ND and HFD libraries. ND, normal diet mice; HFD, high-fat diet mice

In this study, high-throughput sequencing was performed in the liver tissues of each mouse individually (ND, *n* = 5; HFD, *n* = 5). And two small RNAs libraries were constructed from liver tissues of five ND mice and five HFD-induced hepatic IR mice, and subjected to sequence analysis. High-throughput sequencing produced 61,162,625 and 55,472,909 raw reads from the two groups, respectively. After filtering the reads based on basal quality control, a total of 42,006,785 and 37,572,110 clean reads were retained in the ND and HFD groups, respectively (Table 1). The sequences ranged from 18 to 32 nt in length, of which the majority were 21 to 23 nt long, and 22-nt small RNAs were the most abundant in the two libraries (Fig. 2). This is in accordance with a previous finding in other studies of mouse or goat [18, 19]. Meanwhile, the unique reads were obtained by collapsing identical reads for analysis of the small RNA’s species and abundance. The number of unique reads was 2,973,537 in the ND group and 2,363,091 in the HFD group. All unique reads were annotated into different categories of RNAs using the Rfam database (V12.1) and GenBank noncoding RNA database. Conserved miRNAs account for 63.68% (26,749,764 reads) and 65.80% (24,721,306 reads) of total sequence reads, and 4.34% (129,150 reads) and 5.25% (124,042 reads) of the unique sequence reads, in ND and HFD libraries, respectively (Additional file 3).

**Table 1:** Results of raw reads before and after quality control of ND and HFD libraries

Type	ND		HFD	
	Read Number	Percentage(%)	Read Number	Percentage(%)
Raw_reads	61162625	100	55472909	100
Adapter_only	3419341	5.59	3644031	6.57
N_reads	6125	0.01	2011	0.00
<18nt	10039976	16.42	9813990	17.69
>32nt	5690398	9.30	4440767	8.01
Clean_reads	42006785	68.68	37572110	67.73

The first column means the reads types. Raw reads are processed to obtain clean reads, Reads Number means the reads number of each type in ND and HFD libraries. Percentage means the fractional reads number of each type in total reads

### Identification of known and potentially novel miRNAs

**Fig. 3:**
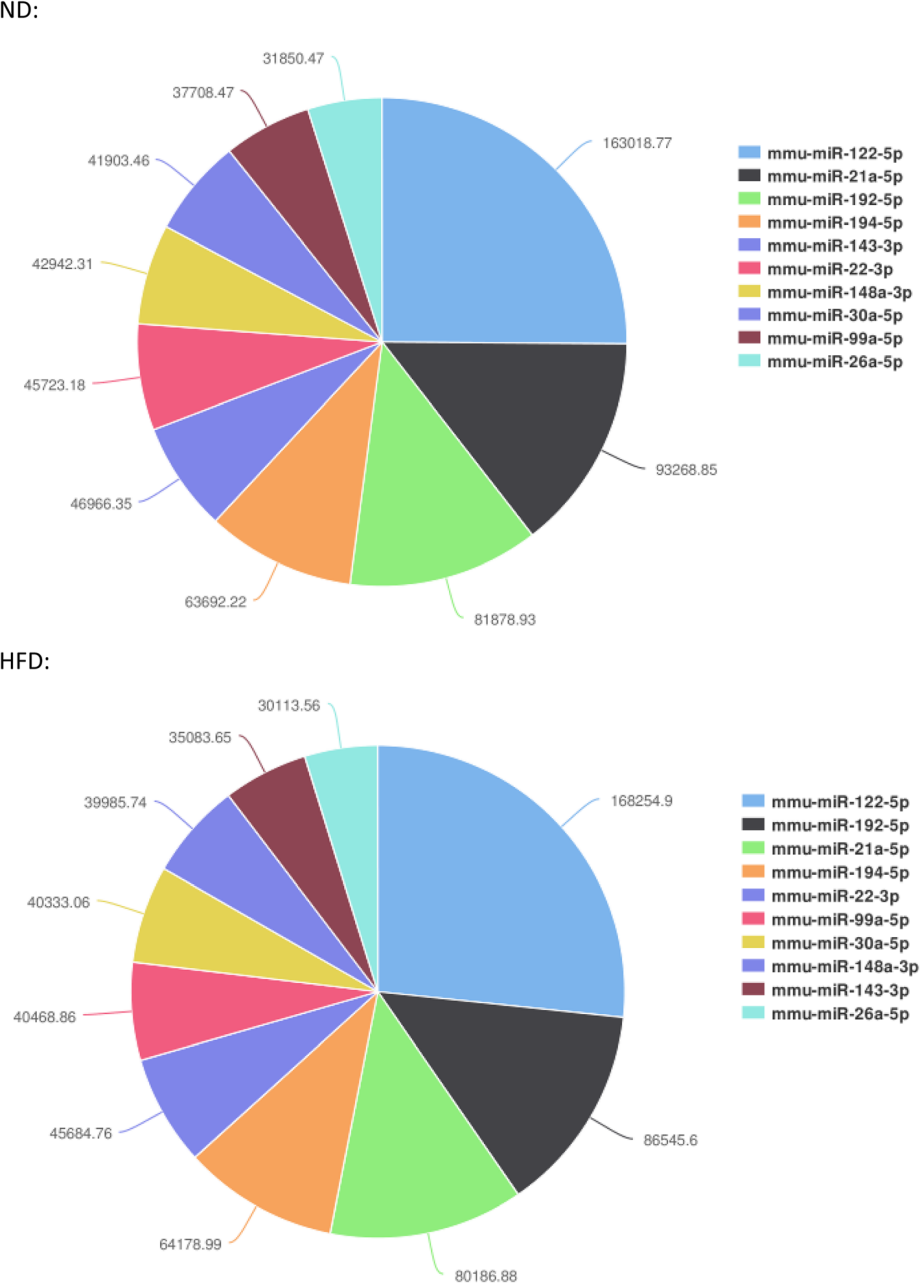
The ten most abundant miRNAs in the two groups. ND, normal diet mice; HFD, high-fat diet mice

In order to identify known miRNAs, the remaining small RNA reads discarding the non-miRNA sequence (tRNA, rRNA, snRNA, etc.) were mapped to the *Mus musculus* data of miRBase 21.0. The expression levels were screened according to a certain standard (counts ≥ 5). A total of 590 known miRNAs were identified, in which 497 miRNAs were co-expressed in both libraries, whereas 56 and 37 miRNAs were specifically expressed in the ND group and HFD group, respectively (Additional file 4). Moreover, the top 10 abundant known miRNAs expressed in two libraries are presented in Fig. 3. In mouse liver tissues, *miR-122-5p* was the most abundant known miRNAs, and the others were *miR-21a-5p*, *miR-192-5p*, *miR-194-5p*, *miR-22-3p*, *miR-143-3p*, *miR-148a-3p*, *miR-30a-5p*, *miR-99a-5p*, and *miR-26a-5p*.

In this study, a total of 395 novel miRNAs were identified from two libraries using miRDeep2 software. Of the 395 novel miRNAs, 374 miRNAs were co-expressed in the two groups, while 16 miRNAs were only expressed in ND library and 5 miRNAs were only expressed in HFD library (Additional file 4).

### Differentially expressed miRNAs between the two groups

**Fig. 4:**
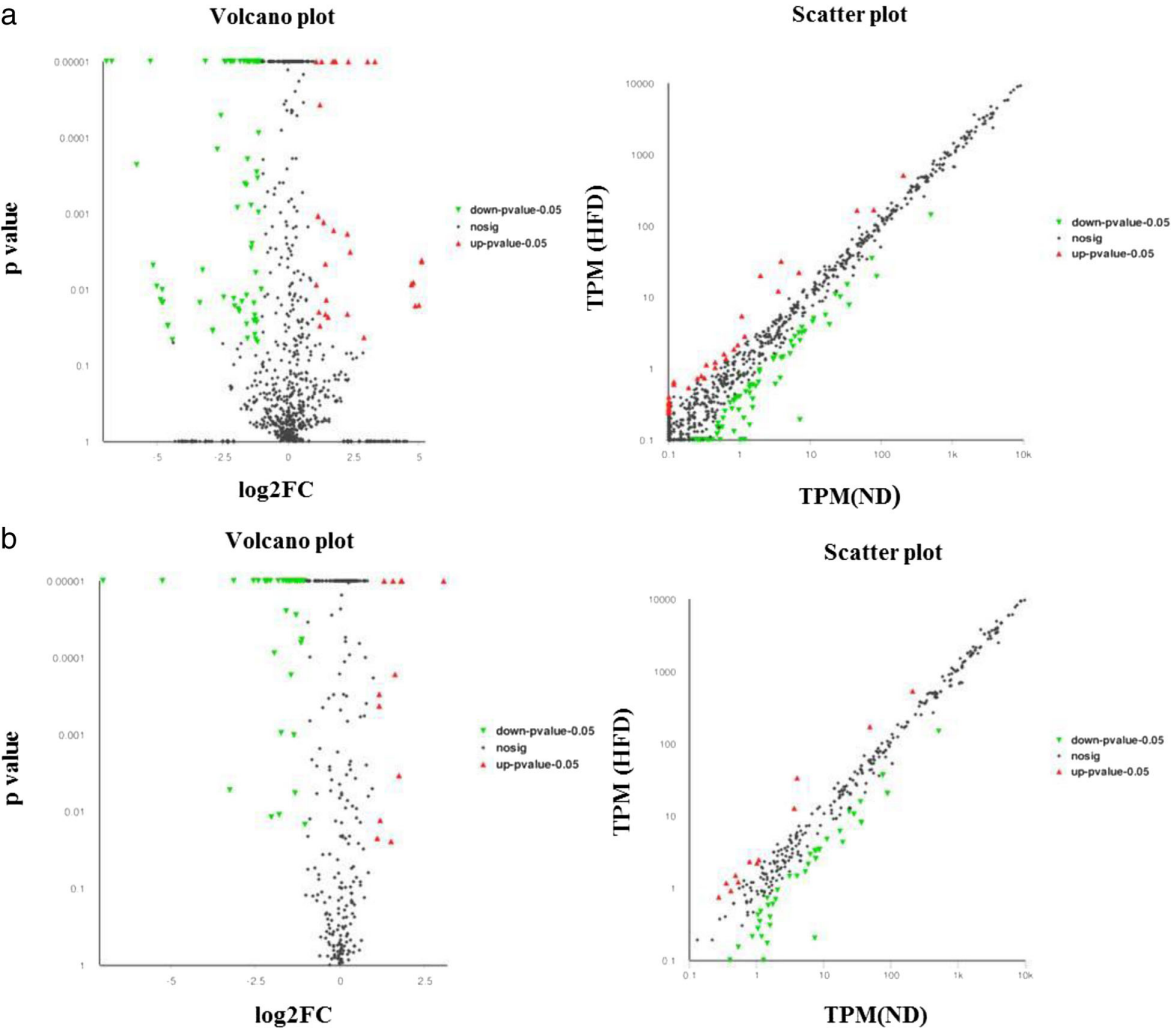
The volcano plot and scatter plot of the known (**a**) and novel (**b**) differentially expressed miRNAs between the two groups. Red spots represent upregulated miRNAs, and green spots indicate downregulated miRNAs. Back spots represent miRNAs that did not show obvious changes between the ND and HFD groups. ND, normal diet mice; HFD, high-fat diet mice

In this study, differential expression analysis showed that 107 known miRNAs and 56 novel miRNAs were identified as differentially expressed between the ND and HFD groups. Compared with the ND group, of 107 differentially expressed known miRNAs, 31 miRNAs were upregulated and 76 miRNAs were downregulated, and in 56 differentially expressed novel miRNAs, 12 miRNAs were upregulated and 44 miRNAs were downregulated in the HFD group (Additional file 5). The volcano plot and the scatter plot were generated to visualize the differentially expressed known and novel miRNAs between the two groups, as shown in Fig. 4. The cluster and heatmaps were plotted to better demonstrate the dysregulated miRNAs between the two groups, as shown in Additional files 6 and 7.

### Prediction of potential targets of differentially expressed known miRNAs, and GO and KEGG analyses

**Fig. 5:**
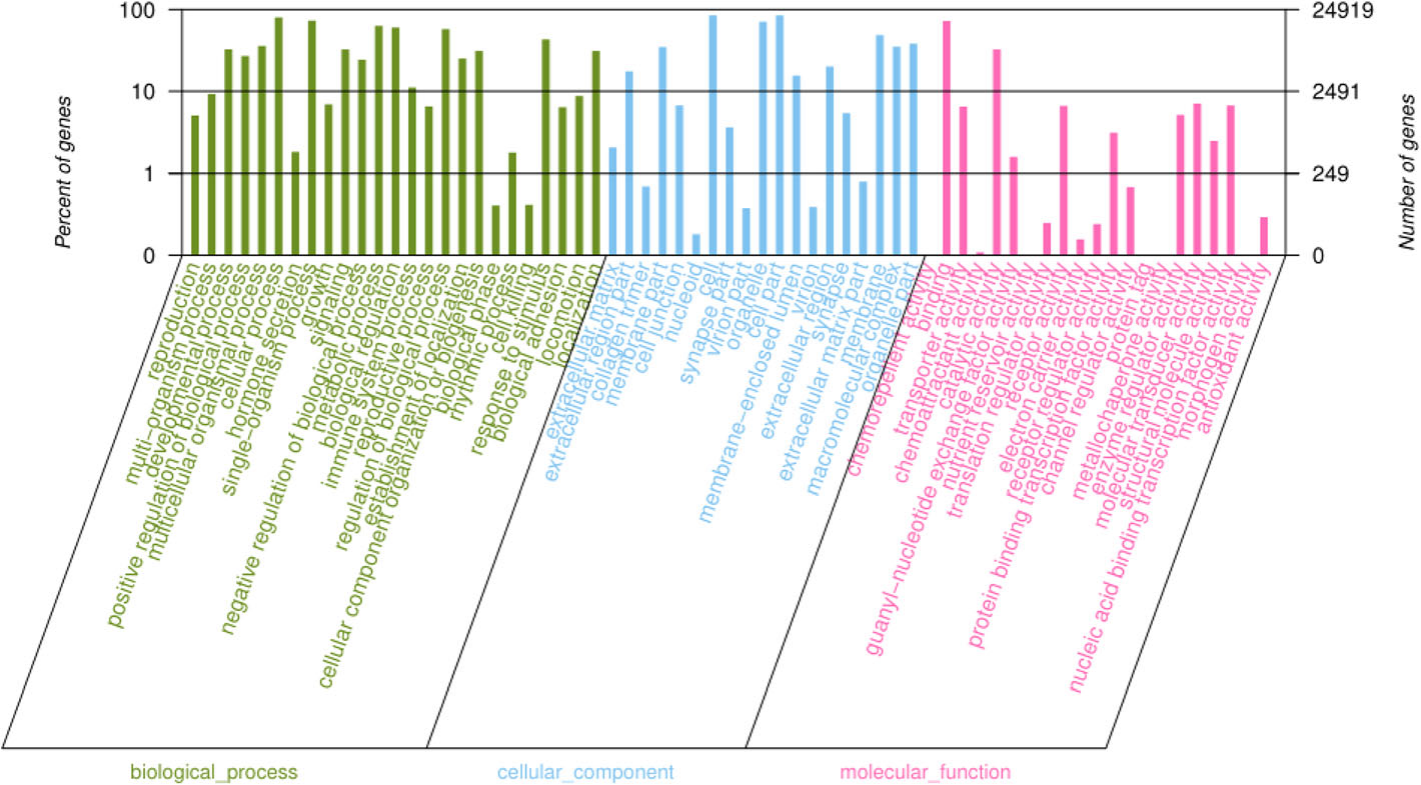
The GO terms of target genes of 50 differentially expressed miRNAs. The χ-axis indicates the subcategories; the left and right y-axis indicates the percent and the number of differentially expressed genes, respectively

**Fig. 6:**
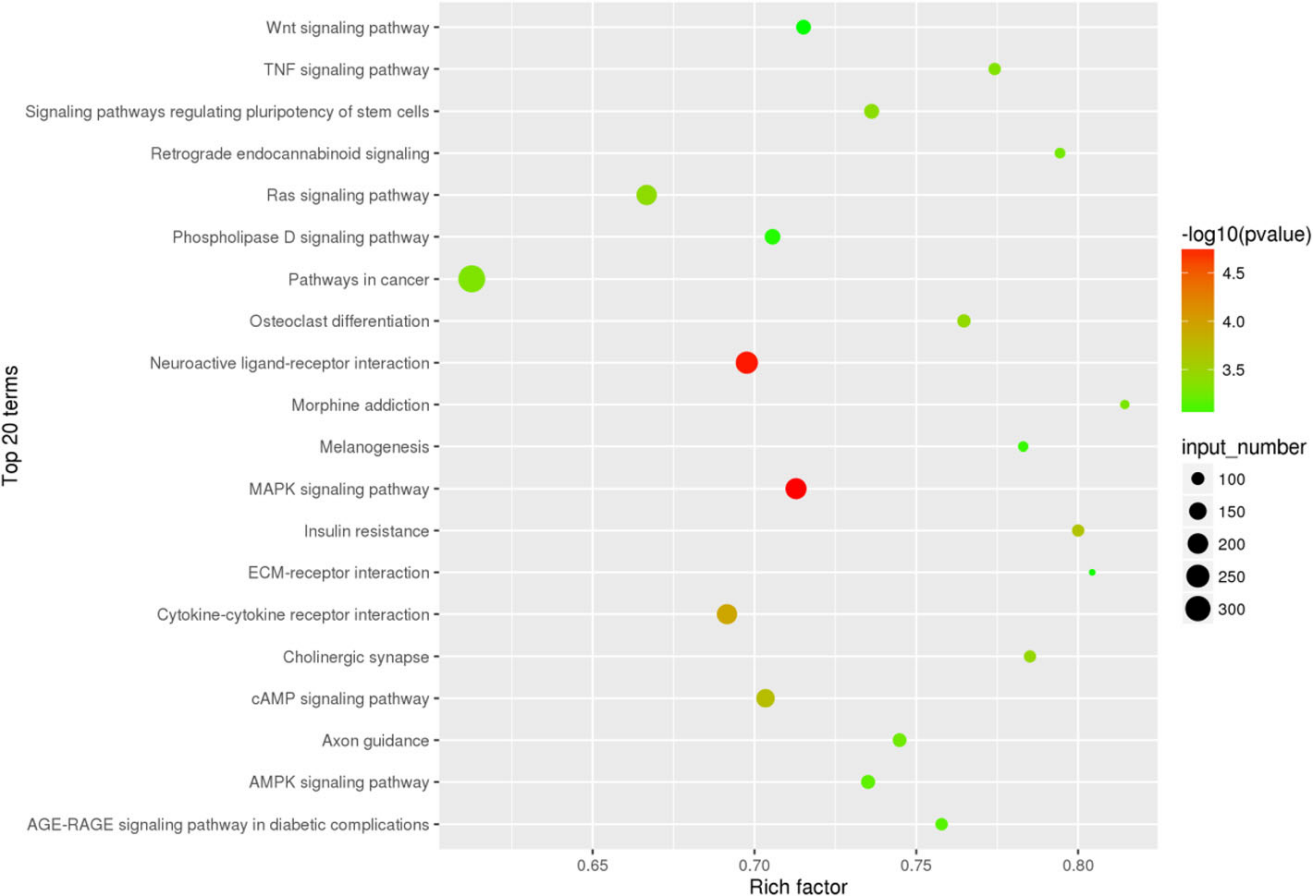
Top 20 KEGG pathways for target gene of differentially expressed miRNAs. The circle size represented gene number. Q value was shown by color gradient

To understand the function of the differentially expressed miRNAs, their target genes were predicted by miRanda. A total of 17,194 target genes were predicted for the 107 known miRNAs. Then, these target genes were performed with GO and KEGG analyses separately. GO categories were assigned to these target genes, according to their cellular components, molecular functions, and biological processes. The target genes of 50 differentially expressed miRNAs, including 9 upregulated and 41 downregulated miRNAs, were analyzed, and GO biological processes associated with metabolic process were found (Fig. 5). KEGG enrichment analysis revealed 315 KEGG pathways were divided into seven classes (environmental information processing, genetic information processing, cellular processes, organismal systems, drug development, human diseases, and metabolism) (Additional file 8). And the top 20 enriched KEGG pathways were showed in Fig. 6, in which the most highly represented group “pathways in cancer,” more importantly, the “insulin resistance” pathway, was also presented.

### Validation of some differentially expressed miRNAs and prediction of their target genes related to IR or glycolipid metabolism

**Fig. 7:**
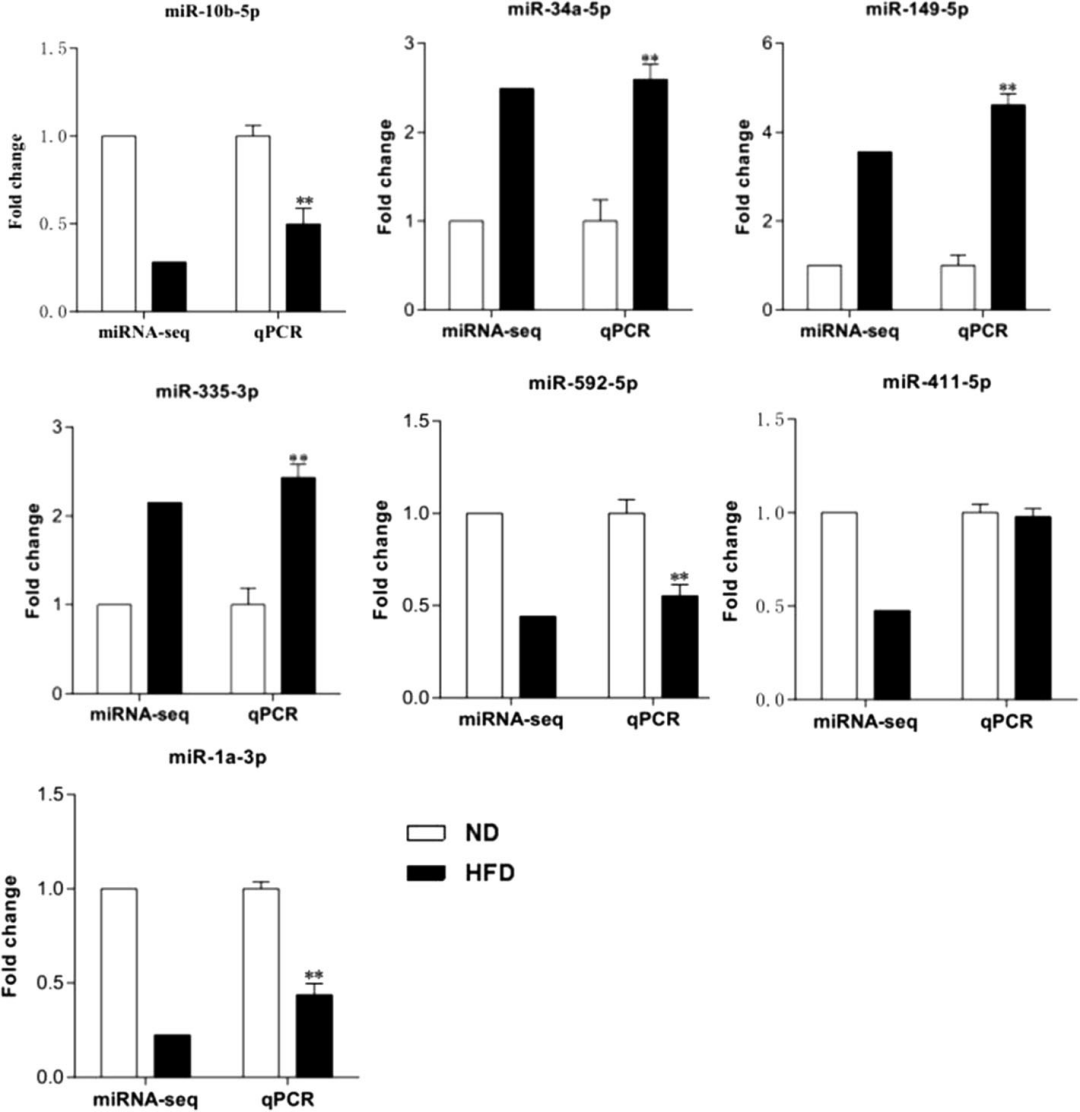
q-PCR validation of some differentially expressed miRNAs identified using high-throughput sequencing in mouse liver tissues. ND, normal diet mice; HFD, high-fat diet mice. *P < 0.05, **P < 0.01, compared with the value of the ND group

In order to validate the reliability of miRNA expression data, seven miRNAs which have relative abundant expression and are significantly differentially expressed in the livers of the two groups of mice were selected and analyzed by quantitative real-time PCR, including three upregulated miRNAs (*miR-34a-5p*, *miR-149-5p*, and *miR-335-3p*) and four downregulated ones (*miR-10b-5p*, *miR-1a-3p*, *miR-411-5p*, and *miR-592-5p*). The results showed that, compared with the ND group, the levels of *miR-34a-5p*, *miR-149-5p*, and *miR-335-3p* were significantly increased, while the levels of *miR-10b-5p*, *miR-1a-3p*, and *miR-592-5p* were decreased in the HFD group. No significant difference was observed in the levels of *miR-411-5p* between the two groups (*P* > 0.05) (Fig. 7). Moreover, the potential target genes of these validated differentially expressed miRNAs were predicted and listed in Additional file 9. Meanwhile, partial target genes related to IR or glycolipid metabolism of these miRNAs are presented in Table 2.

**Table 2:** Lists of 6 verified miRNAs and their targets with functions related to glucolipid metabolism or insulin resistance

miRNA	Target numbers	Partial target genes
miR-10b-5p	900	Foxo1 Irs1 Ppp1r3e
miR-34a-5p	2013	Pdk1 Slc27a6 Ptpn1 Slc27a4 Ppp1r3c Ppp2r4 Pik3cb
miR-149-5p	2425	Crtc2 Ptpn11 Insr Stat3 Rps6kb2 Pik3r1
miR-1a-3p	1206	Ppp1r3b Akt2 Creb5
miR-335-3p	1846	Ogt Ppp1r3a Prkcz Mapk10 Rps6kb1 Pik3r5 Ppargc1a
miR-592-5p	1009	Cpt1a Cd36 Pik3cd Prkab2

## Discussion

IR is defined as impaired sensitivity to insulin in its main target organs, including liver, muscle, and adipose tissues. And impaired insulin action in the liver leads to IR characterized by impairment in the ability of insulin to inhibit glucose output, representing the increase in gluconeogenesis and glycogenolysis. In this study, the mouse model of hepatic IR was induced by feeding of mice with HFD. The data of HOMA-IR and IPGTT demonstrated that HFD mice exhibited impaired glucose tolerance. And the expression of several proteins involved in gluconeogenesis and glycogenolysis, such as PCK1, p-GS, and G6PC, were significantly increased in the liver of HFD mice; meanwhile, p-Akt, as a crucial protein in insulin signaling pathway, was also significantly decreased, suggesting hepatic IR occurs in HFD mice.

MiRNAs have been reported to be associated with hepatic IR [5–8]; however, the exact mechanism is not yet clear. In an effort to gain insights into the molecular mechanisms underlying hepatic IR governed by miRNA regulation, we conducted a high-throughput RNA sequencing to evaluate expression profiles of miRNAs in the livers of normal mice and HFD-induced hepatic IR mice. In this study, some miRNAs, such as *miR-122* and *miR-148a*, were abundant miRNAs in the liver tissues of the two groups of mice, which have been also reported to be highly expressed in human and mouse liver tissues in previous studies [20–22], indicating that they may play essential roles for many physiological processes in the liver, although there was no significant difference in their expression between the two groups in our study. Meanwhile, we found that 107 known and 56 novel miRNAs were differentially expressed between the two groups. Not surprisingly, a proportion of differentially expressed miRNAs which were found to be in our study had been previously reported to play a significant role in IR or IR-related diseases. For instance, an increase in *miR-34a-5p* in the human liver is associated with non-alcoholic fatty liver disease severity [23]. Our study further highlights the importance of these differentially expressed miRNAs in liver tissue development and metabolism. Furthermore, to verify the reliability of miRNA sequencing data, seven miRNAs, which are relatively rich in liver tissues and significantly differentially expressed, were selected and detected by q-PCR in the livers of the two groups. And the expression changes of six miRNAs detected by q-PCR were consistent with the sequencing results. In theory, the expression levels of each candidate miRNA need to be validated using q-PCR; therefore, in our study, all differentially expressed miRNAs can only be regarded as a miRNA reference dataset, and further research should be performed to validate the expression of miRNAs of interest in a larger sample.

The target genes of miRNAs are essential for the function of miRNAs. Therefore, the target genes of differently expressed miRNAs were predicted by miRanda. Afterwards, in order to demonstrate the function of these target genes, functional enrichment analysis was performed for GO and KEGG pathways. The GO annotation illustrated that the predicted target genes of differentially expressed miRNAs were mainly classified in biological processes relevant to regulation of biological process, metabolic process, and regulation of cellular process**.** Interestingly, KEGG pathway annotation of target genes presented an IR-related pathway.

## Conclusions

We constructed small RNA libraries in the livers of normal mice and high-fat diet (HFD)-induced hepatic IR mice. And the differentially expressed miRNAs and their predicted target genes were analyzed and characterized. In addition, the functional categories and pathways of the target genes of differentially expressed miRNAs were analyzed by GO category and KEGG pathway database. Our results defined miRNAs expression signature that may lead to hepatic IR in mice, and the data provided a basis for further investigations to explore the effects and underlying mechanisms of the miRNAs and their target genes in the pathogenesis of hepatic IR and related metabolic diseases.

## Additional files

Additional file 1: The ingradient of normal diet(ND) and high-fat diet(HFD). (DOCX 20 kb)

Additional file 2: Details of the primers used for q-PCR of miRNAs. (XLSX 10 kb)

Additional file 3: Different kinds of small RNA distribution in ND and HFD separately. (XLSX 11 kb)

Additional file 4: Venn diagram of known and novel miRNAs in ND and HFD groups. a and b stand for known miRNAs and novel miRNAs, respectively. The blue part represented miRNAs only expressed in ND group, the orange part represented miRNAs only expressed in HFD group, and the gray part represented miRNAs expressed in both ND and HFD groups. ND, normal diet mice; HFD, high-fat diet mice. (DOCX 52 kb)

Additional file 5: The expression of novel miRNAs in ND and HFD groups. (XLSX 20 kb)

Additional file 6: The heatmap of differentially expressed known miRNAs in ND and HFD groups. (PDF 38 kb)

Additional file 7: The heatmap of differentially expressed novel miRNAs in ND and HFD groups. (PDF 32 kb)

Additional file 8: Predicated target genes pathway enrichment. (XLSX 164 kb)

Additional file 9: The target genes of miR-592-5p. (XLSX 820 kb)
