# Supplementary material for: High-throughput sequencing of small RNAs and analysis of differentially expressed microRNAs associated with high-fat diet-induced hepatic insulin resistance in mice
Source: Genes Nutr. 2019 Feb 19;14:6. doi: 10.1186/s12263-019-0630-1 (PMC6379981; doi:10.1186/s12263-019-0630-1)
Supplement: Supplementary file 1 — The ingradient of normal diet(ND) and high-fat diet(HFD). (DOCX 20 kb) [file 12263_2019_630_MOESM1_ESM.docx]

**Additional file 1 The ingradient of normal diet(ND) and high-fat diet(HFD)**

| Ingredients (g/kg) | Normal-fat diet | High-fat diet |
| --- | --- | --- |
| Corn Starch | 495.692 | 290.692 |
| Casein, 30Mesh | 140 | 140 |
| Maltodextrin 10 | 125 | 125 |
| Sucrose | 100 | 100 |
| Soybean oil | 40 | 40 |
| Lard | 0 | 205 |
| Cellulose | 50 | 50 |
| Mineral mix (AIN-93M) | 35 | 35 |
| Vitamin mix (AIN-93M) | 10 | 10 |
| L-cystine | 1.8 | 1.8 |
| Choline bitartrate | 2.5 | 2.5 |
| t-Butylhydroquinone | 0.008 | 0.008 |
