# Supplementary material for: High-throughput sequencing of small RNAs and analysis of differentially expressed microRNAs associated with high-fat diet-induced hepatic insulin resistance in mice
Source: Genes Nutr. 2019 Feb 19;14:6. doi: 10.1186/s12263-019-0630-1 (PMC6379981; doi:10.1186/s12263-019-0630-1)
Supplement: Supplementary file 4 — Venn diagram of known and novel miRNAs in ND and HFD groups. a and b stand for known miRNAs and novel miRNAs, respectively. The blue part represented miRNAs only expressed in ND group, the orange part represented miRNAs only expressed in HFD group, and the gray part represented miRNAs expressed in both ND and HFD groups. ND, normal diet mice; HFD, high-fat diet mice. (DOCX 52 kb) [file 12263_2019_630_MOESM4_ESM.docx]

**Additional file 4.** Venn diagram of known and novel miRNAs in ND and HFD groups.

a


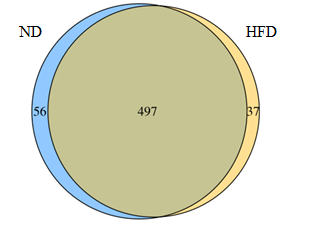


b


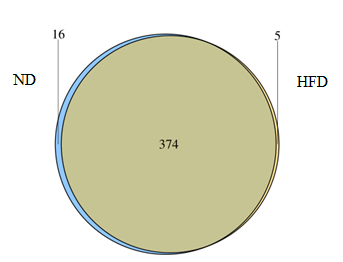


a and b stand for known miRNAs and novel miRNAs, respectively. The blue part represented miRNAs only expressed in ND group, the orange part represented miRNAs only expressed in HFD group, and the gray part represented miRNAs expressed in both ND and HFD groups. ND, normal diet mice; HFD, high-fat diet mice.
