# Supplementary figures and images for: High-throughput sequencing of small RNAs and analysis of differentially expressed microRNAs associated with high-fat diet-induced hepatic insulin resistance in mice
Source: Genes Nutr. 2019 Feb 19;14:6. doi: 10.1186/s12263-019-0630-1 (PMC6379981; doi:10.1186/s12263-019-0630-1)

Color Key

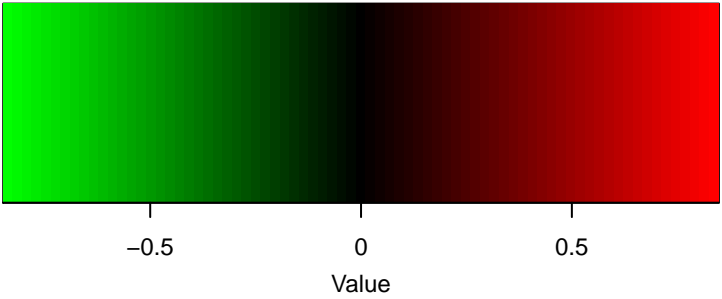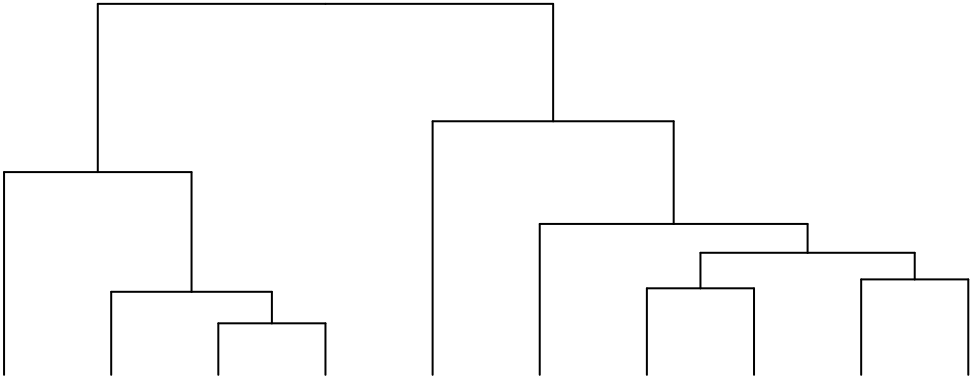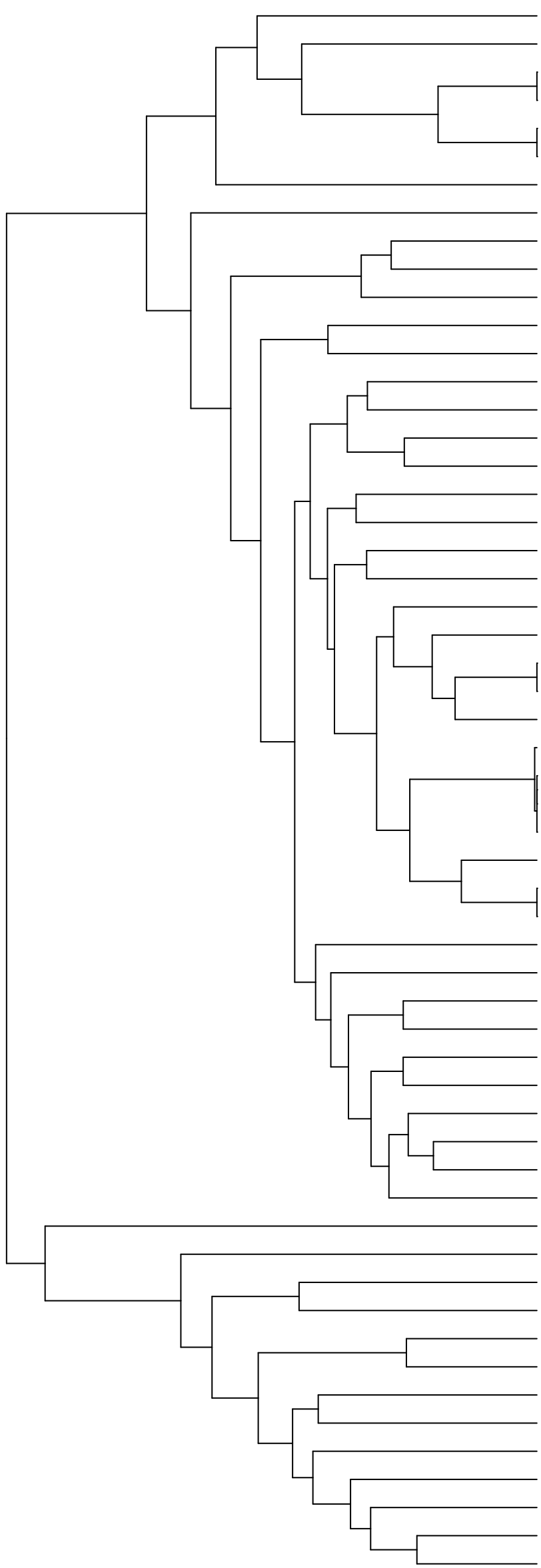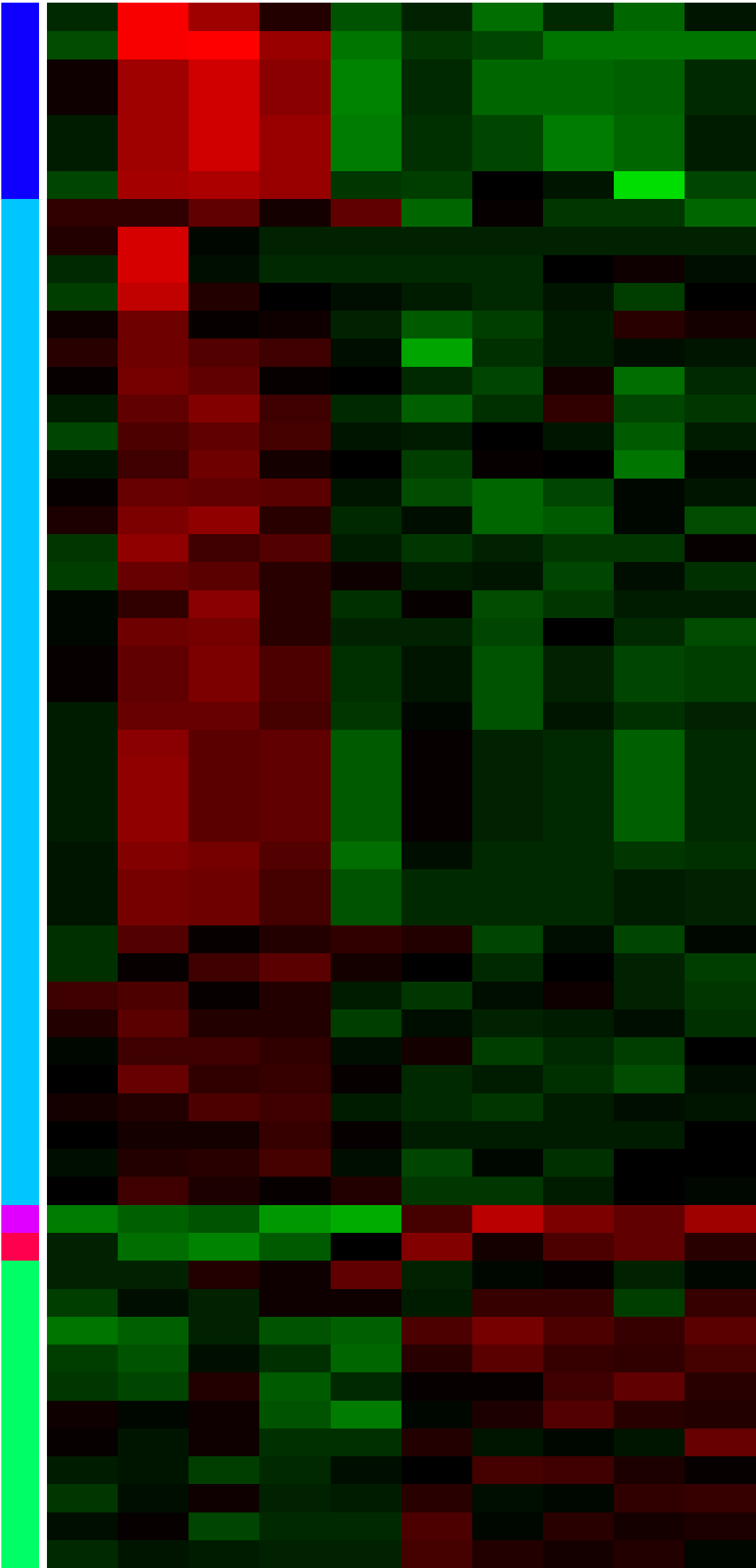

Supplement: Supplementary file 7 — The heatmap of differentially expressed novel miRNAs in ND and HFD groups. (PDF 32 kb) [file 12263_2019_630_MOESM7_ESM.pdf]
